# Physical properties and lung deposition of particles emitted from five major indoor sources

**DOI:** 10.1007/s11869-016-0424-1

**Published:** 2016-08-25

**Authors:** Tuan V. Vu, Jakub Ondracek, Vladimir Zdímal, Jaroslav Schwarz, Juana Maria Delgado-Saborit, Roy M. Harrison

**Affiliations:** 1 Division of Environmental Health & Risk Management, School of Geography, Earth & Environmental Sciences, University of Birmingham, Edgbaston, Birmingham B15 2TT UK; 2 Institute of Chemical Process Fundamentals of the ASCR (ICPF), Prague, 165 02 Czech Republic; 3 Department of Environmental Sciences/Center of Excellence in Environmental Studies, King Abdulaziz University, PO Box 80203, Jeddah, 21589 Saudi Arabia

**Keywords:** Indoor sources, Particle size, Hygroscopic growth, Lung deposition

## Abstract

The physical properties of indoor particles were measured with an Scanning Mobility Particle Sizer (SMPS) system (14.6–850 nm), an Aerodynamic Particle Sizer (APS, 0.54–18 μm) and an Hygroscopic Tandem Differential Mobility Analyzer (H-TDMA) in an apartment located in an urban background site in Prague (Czech Republic) from 15 August to 8 September, 2014. The total particle maximum number concentration was 9.38 × 10^4^, 1.46 × 10^5^, 2.89 × 10^4^, 2.25 × 10^5^ and 1.57 × 10^6^ particles cm^−3^ for particles released from vacuum cleaning, soap/W5 cleaning spray, smoking, incense burning and cooking (frying) activities, respectively. Particles emitted from cleaning activities showed unimodal number size distributions, with the majority of particles (>98.2 %) in the ultrafine size range (Dp <100 nm) and modes at a diameter of 19.8 nm for vacuum cleaning and 30.6 nm for soap/W5 cleaning. Smoking and incense burning predominantly generated particles in the accumulation mode with a count median diameter around 90–150 nm while cooking emissions showed a bimodal structure with a main mode at 47.8 nm. Particles from vacuum cleaning, incense burning, smoking and cooking emissions were found to be “nearly hydrophobic” with an average growth factor (G_f_) around 1.01–1.10, while particles emitted from desk cleaning using organic compounds were found to be “less-hygroscopic” (G_f_ ∼1.12–1.16). Based on an adjusted MPPD model with a consideration of the hygroscopic properties of particles, the total lung deposition fractions of these particles by number when they penetrate into the human lung were 0.73 ± 0.02, 0.62 ± 0.03, 0.37 ± 0.03, 0.32 ± 0.03 and 0.49 ± 0.02 for vacuum cleaning, desk cleaning, smoking, incense burning and cooking, respectively.

## Introduction

People in developed countries spend the majority of their time (approximately 90 %) in indoor environments (Delgado-Saborit et al. 2011), and as consequence they may be exposed to a range of pollutants of an indoor origin, particularly ultrafine particles which may cause cardiovascular, respiratory, and neurological hazards to human health (Diffey 2011; Donaldson et al. 1998). Morawska et al. (2013) reported that 19–76 % of the integrated daily residential exposure to ultrafine particles originated from indoor-generated particles.

In recent years, many studies have been performed to characterize a range of indoor-generated particles from various microenvironments such as home, office, school or work place. For example, He et al. (2004) measured the contribution from 21 different types of indoor activities to particle number and mass concentration in 15 residential houses. Their study found that the indoor particle number concentrations showed an increase by 1.5 to over 27 times during the indoor activities, while the PM_2.5_ concentration was also estimated to increase during smoking, grilling and frying activities from 3 to 90 times above the background level. Similarly, Bhangar et al. (2011) investigated ultrafine particle concentration in seven residences in northern California and indicated that cooking was the most important indoor activity contributing to the indoor ultrafine particle level.

Different types of indoor activities release particles with different physical properties including their size distribution. The majority of particles generated from indoor combustion sources including cooking, wood burning, candle burning, fireplace or kerosene heating were found in the submicron size range (Hussein et al. 2006). On the other hand, particles originating from resuspension from indoor surfaces due to building occupant movement are predominantly distributed in the coarse mode, with a diameter lager than 1 μm (Thatcher and Layton 1995). Characterization of the particle size distribution of indoor sources is not only useful in determining the regional lung deposition of particles, but also in source apportionment of indoor aerosols based on receptor modelling methods (Vu et al. 2015b). Ogulei et al. (2006) ran a positive matrix factorization (PMF) analysis on indoor particle size distributions in an occupied townhouse in Reston (Washington, DC, USA) to identify the contribution of indoor sources to indoor aerosols.

Although the number of studies focusing on indoor aerosols has increased in recent years, the physical characterization database of indoor sources, particularly the hygroscopicity of indoor particles which is known to be an important determinant of lung deposition fraction of particles in the human respiratory tract, is still limited (Vu et al. 2015a). The aim of this study was to investigate physical properties including size distribution, effective density and hygroscopicity of particles originating from five typical indoor sources. The regional lung deposition fraction of indoor particles was calculated based on a modified Multiple-Path Particle Dosimetry Model (MPPD). Finally, the minute regional lung dose of indoor-generated particles was estimated and compared.

## Materials and methods

### Site description and data measurement

**Fig. 1:**
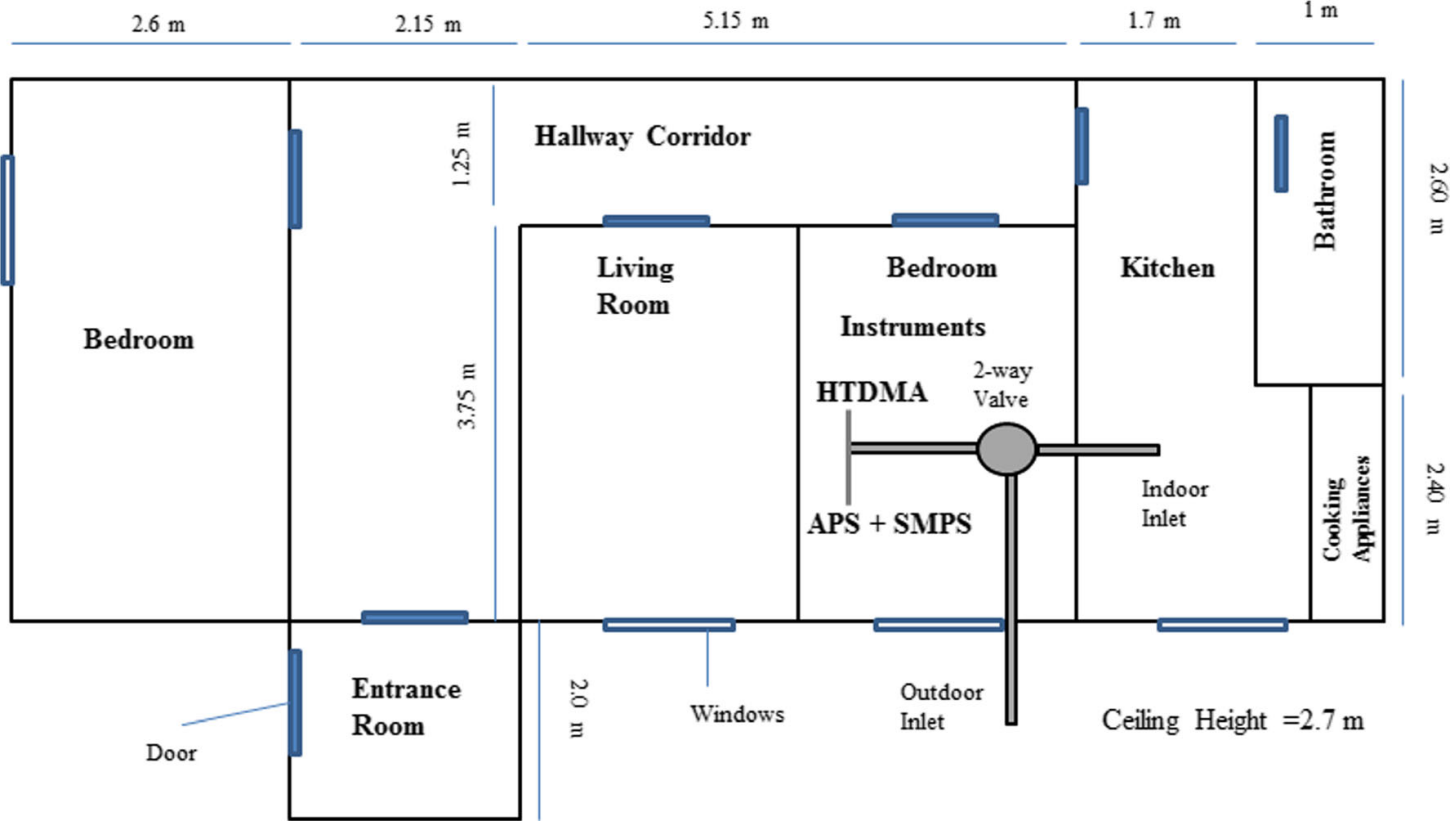
Plan view of the apartment

The experimental campaign was conducted from 15 August to 8 September, 2014 in an apartment located in the suburban background of Prague, Czech Republic. The apartment was unoccupied during the sampling period. It is located on the ground floor of a two-floor building and has a living room, a small bathroom next to the kitchen and two bedrooms, one of them containing the instruments. The apartment (as shown in Fig. 1) and sampling site have been described in detail by Hussein et al. (2006).

The particle number size distribution (14.6–850 nm) was measured by a Scanning Mobility Particle Sizer system (SMPS 3696, TSI Inc., USA) comprising a TSI 3080 electrostatic classifier, a TSI 3081 differential mobility analyzer (long DMA) and a TSI 3775 condensation particle counter (CPC). Larger particles and their number size distribution (0.54–18 μm) were measured by an Aerodynamic Particle Sizer (APS 3321, TSI Inc., USA). The SMPS system was operated at low aerosol flow rate of 0.3 L min^−1^ and the APS flow rate was 5 L min^−1^, with 5 min time resolution. A Hygroscopic Tandem Differential Mobility Analyzer (HTDMA) developed by the Laboratory of Aerosol Chemistry and Physics, Institute of Chemical Process Fundamentals of the ASCR (ICPF, Czech Republic) was installed to measure the hygroscopic growth factors at 90 % relative humidity for particles with three selected initial dry sizes at diameters of 50, 100 and 200 nm.

Indoor particles were generated in a closed kitchen, whose dimensions are shown in Fig. 1. The cleaning, smoking, incense burning and cooking activities were conducted during a period of approximately 10, 5, 60 and 20 min, respectively. Smoking experiments were conducted by two persons who smoked with two cigarettes inside the kitchen. Cooking particles were generated by frying sausages with sunflower oil, and toasting bread. The indoor concentration was at a background level before generating indoor sources. The sampling inlet was put in the breathing zone. All SMPS/APS datasets were corrected for particle loss inside the inlet tube before data analysis.

### Data handling

#### Merging SMPS/APS data

Two types of data sets (aerodynamic and mobility) collected from APS and SMPS instruments were merged into one particle size spectrum matrix (mobility diameter from 0.015 to 10 μm) using an enhanced algorithm which was developed in CRAN R by Beddows et al. (2010). The effective density of particles was estimated based on the best fit between two instrument (APS/SMPS) datasets for number, surface area and volume spectra. The final diameter type obtained by this enhanced merging algorithm was mobility diameter. This type of diameter may be converted to aerodynamic diameter using the following equation:

1 $$ {\mathrm{D}}_{\mathrm{a}}=\mathrm{x}.{\mathrm{D}}_{\mathrm{m}}.\sqrt{\frac{\mathrm{C}\left({\mathrm{D}}_{\mathrm{m}}\right)}{\mathrm{C}\left({\mathrm{D}}_{\mathrm{a}}\right)}} $$

2 $$ \mathrm{x}=\sqrt{\frac{\rho_e}{\rho_o}} $$

where, *D*_a_, *D*_m_ are aerodynamic and mobility diameters (nm), respectively, *C* is the Cunningham slip correction factor and *x* is known as the free parameter that is determined by giving the best fit between APS/SMPS spectra. *ρ*_0_ is unit density and *ρ*_e_ is the estimated transition-regime effective density (g cm^−3^). PM mass concentrations were estimated from the merged size distribution and effective density. In this study, we assume particles are spherical; therefore, their mobility diameters are equal to their equivalent volume diameter. Mobility diameter was used as the input diameter in the MPPD model.

#### Estimation of hygroscopic growth factors in regions of the lung

To analyse the growth factors from the HTDMA, a TDMAinv conversion approach provided by the Laboratory of Atmospheric Chemistry, Paul Scherrer Institute (PSI, Switzerland) was applied (Gysel et al. 2009). This TDMAinv toolkit was run on the Igor Wave Metric software version 6.1. This algorithm retrieved the actual growth factor probability density function (G_f_-PDF) as a piecewise linear function from the measurement distribution function of the HTDMA. The G_f_-PDF is defined as the growth factor probability density function, *c*(*g*,*D*), for particles with dry diameter *D* = *D*_0_ to present a growth factor (G_f_) = *g*, and the total probability of the presented G_f_ is unity: ∫_0_^∞^ *c*(*g*, *D*_0_)*dg* = 1 (Gysel et al. 2009). In this study, the HTDMA measured the mean growth factor at 90 % RH: G_f mean_ = ∫_0_^∞^ *gc*(*g*, *D*_0_)*dg*.

To calculate the growth factors at 99.5 % RH, which is assumed for the RH in the respiratory tract, from the observed growth factor at 90 % RH from our TDMA measurements, we applied an approach provided by Rissler et al. (2010) using the following calculation:

3 $$ {\mathrm{G}}_{\mathrm{f}-99.5\%}{=}^3\sqrt{1+\frac{99.5}{90}\left({G}_{f-90\%}^3-1\right)\frac{\left({C}_{k\kern0.5em \mathrm{at}\kern0.5em 90\%\kern0.5em RH}-0.90\right)}{\left({C}_{k\kern0.5em \mathrm{at}\kern0.5em 99.5\%\kern0.5em RH}-0.95\right)}} $$

where *C*_k_ is the Kelvin curvature correction factor:

4 $$ {C}_{\mathrm{k}\kern0.5em  at\kern0.5em a\%\kern0.5em RH}= \exp \left(\frac{4{\mathrm{M}}_{\mathrm{W}}{\sigma}_{\mathrm{s}}}{\mathrm{RT}.{\uprho}_{\mathrm{w}}{\mathrm{D}}_{\mathrm{p}-\mathrm{at}\kern0.5em \mathrm{a}\%\kern0.5em \mathrm{R}\mathrm{H}}}\right) $$

*M*_W_ (18 g mol^-1^) and *ρ*_w_ (1 g cm^−3^) are the molecular weight and density of water; *σ*_s_ is the surface tension of the solution (assuming a surface tension of 0.072 J m^−2^); *R* (8.314 J mol^−1^) and *T* (298 K) are the ideal gas constant and temperature, respectively, and *D*_p_ is the particle diameter (nm).

In this study, the HTMA only measured particles at three selected dry sizes of 50, 100 and 200 nm at 90 % RH. For particles of below 50 nm and over 200 nm, this study assumed that the soluble volume fraction of particles at a diameter of 50 nm is also descriptive of those smaller than 50 nm while those of particles with a diameter of 200 nm also describe those larger than 200 nm. In our measurements, particle size distributions were collected using SMPS/APS instruments without a dryer. It is therefore necessary to calculate the effects of ambient humidity on the size distribution measured by the SMPS/APS before using the data for the lung dose calculation. The dry diameter of a particle can be estimated by the ratio of particle size measured by SMPS and the growth factor of that dry particle in the ambient relative humidity (RH). The RH for the indoor environment during vacuum cleaning, soap cleaning, smoking, incense burning and cooking was 47.9, 57.2, 47.3, 66.21 and 72.8 %, respectively. According to our calculation, the effect of ambient relative humidity on the measured size distributions is not significant since the growth factors of particles were approximately unity under these low RH conditions.

When a particle penetrates into the lung, its growth not only depends on particle size but also upon its residence time. To address the particle growth dependence upon time, we used an approach provided by Ferron (1977):

5 $$ \mathrm{F}\left(\mathrm{t}\right)=\frac{\mathrm{Dp}\left(\mathrm{t}\right)-\mathrm{D}\mathrm{p}(0)}{\mathrm{Dp}\left(\mathrm{e}\right)-\mathrm{D}\mathrm{p}(0)}=\frac{\mathrm{Dp}(0)\times \left[ exp{\left(-\frac{10{\mathrm{t}}^{0.55}}{\mathrm{Dp}(0)}\right)}^{0.6}-1\right]}{\mathrm{Dp}\left(\mathrm{e}\right)-\mathrm{D}\mathrm{p}(0)} $$

where *F*(*t*) is fraction of particles at equilibrium size, Dp(*t*) is particle diameter at time *t* (s); Dp(0) is the initial dry particle diameter (μm), Dp(e) is equilibrium particle diameter (μm) and *t* in the residence time in the lung(s).

Therefore, the particle growth factor in each region will be calculated by:

6 $$ \mathrm{Averaged}\kern0.5em \mathrm{G}\mathrm{f}\kern0.5em \mathrm{in}\kern0.5em \mathrm{each}\kern0.5em \mathrm{lung}\kern0.5em \mathrm{region}=\frac{{\displaystyle {\int}_{{\mathrm{t}}_1}^{{\mathrm{t}}_2}\frac{\mathrm{Dp}\left(\mathrm{t}\right)}{\mathrm{Dp}(0)}\mathrm{d}\mathrm{t}}}{{\mathrm{t}}_2-{\mathrm{t}}_1} $$

where *t*_1_ and *t*_2_ are the time when the particle enters and leaves the region of the lung.

### Modelling particle deposition in the human respiratory system

Many mathematical models have been developed to estimate the total and regional lung deposition of particles in recent decades; for example the ICRP model (International Commission on Radiological Protection), the NCRP model (National Council on Radiation Protection and Measurement), the IDEAL model (Inhalation, Deposition and Exhalation of Aerosols in/from the Lung) or the MPPD model (Multiple-Path Particle Dosimetry) (Asgharian et al. 2001; ICRP and Protection 1994; Koblinger and Hofmann 1990; National Council on Radiation Protection and Measurements 1997). This study utilized the MPPD model to calculate the total and regional lung deposition of particles emitted from each source.

The MPPD model was developed by Asgharian et al. (2001). The software (MPPD software version 2.11) was downloaded via http://www.ara.com/products/mppd.htm. In this study, we used the MPPD model to calculate the deposition fraction of particles by number in three regions of the lung, the extra-thoracic (ET), tracheo-bronchial (TB) and alveolar (AL) regions, and the entire lung for adults. This study used the reference respiratory values for light exercise for Caucasian people during cooking and cleaning activities and for resting (sitting) during smoking and incense burning based on the reference data recommended by ICRP (1994). The input data for the MPPD model are shown in Table 1.

**Table 1:** Input data to the MPPD model based on the reference respiratory values from ICRP (1994)

MPPD model input data		Cooking, cleaning		Smoking, incense burning	
		Man	Woman	Man	Woman
	Model	Yeh/Schum 5-Lobe			
	Functional residual capacity (mL)	3301	2681	3301	2681
	Upper respiratory tract volume (mL)	50	50	50	50
Particle properties	Density (g cm^−3^)	0.88–1.56 (by APS/SMPS merging)			
	Nanoparticle model	YES (for particle smaller than 100 nm)			
	Inhalability adjustment	YES (for particle larger than 1 μm)			
	Geometric standard deviation	1			
Exposure scenario	Acceleration of gravity (cm s^−2^)	981	981	981	981
	Body orientation	Upright			
	Breathing frequency (min^−1^)	20	21	12	14
	Tidal volume (mL)	1250	992	750	464
	Inspiratory fraction	0.435			
	Pause fraction	0.05			
	Breathing scenario	Nasal	Nasal	Nasal	Nasal

However, the MPPD model may under-estimate the lung deposition of ambient particles if their hygroscopic properties are not taken into account (Vu et al. 2015a). For correction for the influence of particle hygroscopicity, the MPPD curves were modified for both indoor and outdoor particles with a consideration of their hygroscopicity according to an approach introduced by previous studies (Kristensson et al. 2013; Löndahl et al. 2009; Vu et al. 2015a). In this approach, submicron particles which have increased to their equilibrium size by their growth in the regional lung have the same deposition fraction in the respiratory system as hydrophobic particles with an identical size. Because of no information being available for the hygroscopic growth factor for coarse particles, this study only calculated the deposition fraction of submicron particles (Dp <1 μm) by number. However, the deposition fraction of submicron particles by number is not significantly different to that of total particles since the submicron particles accounted for more than 99 % of total particles by number.

## Results and discussion

### Particle size distributions

#### Outdoor/indoor background levels

**Fig. 2:**
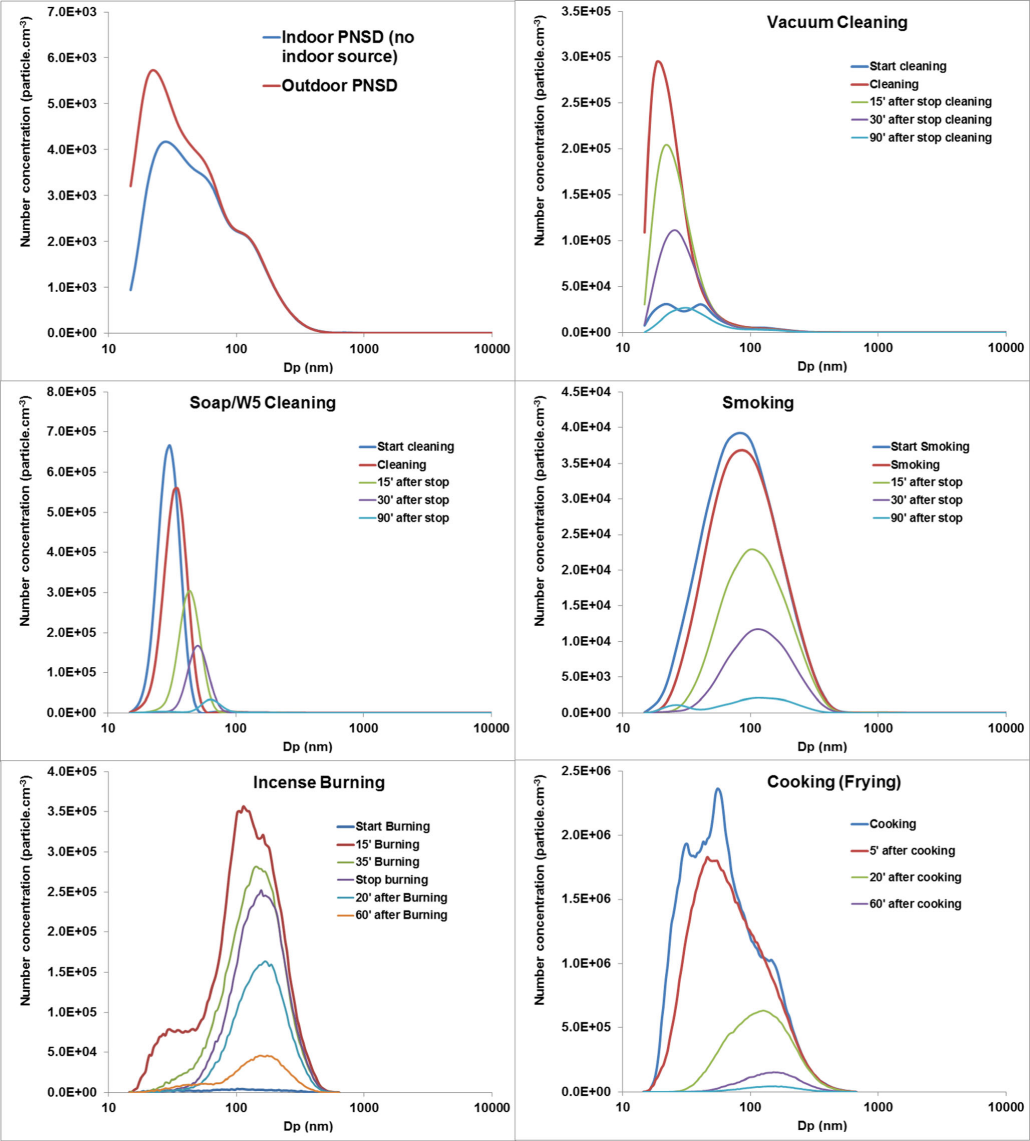
Particle number size distribution from indoors (without indoor sources) and outdoor environments and five major indoor sources

The total number concentration for outdoor and indoor levels (with no indoor sources) was 4.2 ± 2.1 × 10^3^ and 3.3 ± 1.3 × 10^3^ particles cm^−3^, respectively. As shown in Fig. 2, the particle number size distribution of both outdoor and indoor particles appears to be the sum of log-normal size modes. The number mode for outdoor and indoor particle size distributions was 22.9 and 28.4 nm, respectively. The majority of particles by number (84.0 and 80.2 % of total outdoor and indoor particles, respectively) was found in the ultrafine size range (Dp <100 nm).

#### Vacuum cleaning

The peak particle number concentration was 9.4 × 10^4^ particles cm^−3^, which decreased to 7.08 × 10^4^ particles cm^−3^ by the 15th minute after cleaning stopped. The particle number size distribution shows a unimodal distribution with the mode at 19.8 nm. This mode increased to 22.9 and 26.5 nm at 15 and 30 min after the activity stopped. More than 98 % of total particles by number was found in the ultrafine particle size range (Dp <100 nm) during vacuum cleaning. This high number of ultrafine particles emitted from the vacuum cleaner is consistent with previous studies (Géhin et al. 2008; Knibbs et al. 2011; Wu et al. 2011). Knibbs et al. (2011) investigated particle emissions from 21 vacuum cleaners in a flow tunnel and found that the median emission rate of ultrafine particles was 9.92 × 10^9^ particles cm^−3^ with a median value of count median diameter (CMD) of 25.5 nm.

In term of mass concentration, vacuum cleaning also generated a large fraction of coarse particles. The peak PM_1.0_, PM_2.5_ and PM_10_ mass concentrations were 1.5, 22.7 and 75.4 μg m^−3^. Szymczak et al. (2007) suggested that particles are in part generated by mechanical abrasion of the graphite brushes and copper commutator. However, the major source of ultrafine particles may be due to spark discharging that occurs at voltages above 100 V between two carbon electrodes within the vacuum cleaner motor (Helsper et al. 1993; Szymczak et al. 2007). Knibbs et al. (2011) reported lower ultrafine particle emissions with two battery-driven vacuum cleaners at lower voltages (14 and 22 V).

There are few studies on the chemical properties and morphology of particles emitted from vacuum cleaners. Szymczak et al. (2007) used a MOUDI to collect particles in the size range of 0.057–18.0 μm and suggested that ultrafine particles comprise mainly of copper which may be generated from abrasion of the copper commutator inside the motor. Lioy et al. (1999) reported that particles larger than 0.01 μm in diameter mainly consist of chemical binders, copper and carbon (elemental and organic) which were induced by rubbing and arcing between carbon rods and the copper commutator. In this study, the effective density of vacuum cleaner-generated particles obtained by the APS/SMPS merging algorithm was 1.16 g cm^−3^ which was much lower than the material density of carbon (∼2 g cm^−3^) and copper (8 g cm^−3^). This finding suggests that particles released from the vacuum cleaner motor were possibly carbon internal void aggregates (DeCarlo et al. 2004; Helsper et al. 1993; Lioy et al. 1999) which have a lower effective density.

#### Kitchen cleaning by soap/W5 spray cleaner

As seen in Fig. 2, kitchen desk cleaning using organic compounds (brand name, W5 cleaner) generated predominantly ultrafine particles with a maximum concentration of 1.25 × 10^5^ particle cm^−3^ and a peak number mode of 30.6 nm. As with the vacuum cleaning, the CMD increased and particle number concentration decreased rapidly due to coagulation and deposition processes after the cleaning activity was finished.

Cleaning using chemical cleaners also generated both ultrafine and coarse particles. Average PM_1.0_, PM_2.5_ and PM_10_ mass concentration during cleaning activity were 2.5, 10.4 and 22.0 μg m^−3^, respectively. On the other hand, nano/ultrafine particles were probably produced by the oxidation and condensation of volatile organic compounds (VOCs) released from the cleaning agent during the cleaning activity (Nazaroff and Weschler 2004; Rohr 2013; Singer et al. 2006a; Zhu et al. 2001). Singer et al. (2006b) reported that a large amount of VOCs were found using pine-oil cleaner. They reported a concentration measured over 1 h of 10–1300 μg m^−3^ for individual terpenoids, including α-terpinene (90–120 μg m^−3^), d-limonene (1000–1100 μg m^−3^), terpinolene (900–1300 μg m^−3^) and α-terpineol (260–700 μg m^−3^). In addition, Sarwar et al. (2004) found that terpenes from cleaning products can react with ozone, resulting in secondary organic aerosol production in an indoor environment.

This study used W5 orange cleaner, which contains some surface active components (not specified in the detail from the product’s label), soap and limonene. In general, limonene has been identified to play an important role in the formation of indoor nanoparticles (Langer et al. 2008; Wainman et al. 2000; Wang et al. 2007b; Waring et al. 2011). Langer et al. (2008) showed that the nucleation and growth of particles from the reaction of O_3_ and limonene could occur even at low concentration of reactants. The effective density obtained by the APS/SMPS merging process was 0.88 g cm^−3^, indicating that the majority of particles generated by use of cleaning products were predominantly organic.

#### Tobacco smoking

The particle number size distribution from cigarette smoking showed a unimodal structure with a mode at 90 nm and peak number concentration of 2.89 × 10^4^ particle cm^−3^ (Note that two cigarettes were simultaneously smoked by regular smokers in this experiment). During the ageing process, the CMD increased to 120 nm, as shown in Fig. 2. These results are consistent with previous studies (Hussein et al. 2006; Wu et al. 2011). Wu et al. (2011) measured the submicron particle number size distribution emitted from five brands of cigarettes and found that the number mode ranged from 102.9 to 116.7 nm with the maximum number concentration of 1.38 × 10^6^ particle cm^−3^. The number concentration of particles is not only dependent on the emission rate of the source, but also the volume of the chamber or the indoor environment where smoking takes place, and the ventilation.

Fine particles (Dp <2.5 μm) emitted from cigarette smoking were found to make a dominant contribution to mass concentration with an averaged fraction of more than 82 % of total PM_10_ mass. Averaged PM_1.0_, PM_2.5_ and PM_10_ concentrations were 3.2, 133.6 and 149.6 μg m^−3^, respectively. In a review of indoor particles, Wallace (1996) indicated that the most important indoor source of fine and coarse particles in the USA was tobacco smoking, with an estimated increase of up to 45 μg m^−3^ in homes with smokers. The effective density of cigarette smoke particles was 1.56 g cm^−3^ based on APS/SMPS merging results. This value was slightly lower than the material density of black carbon (∼2 g cm^−3^) and similar to the effective density of humic acids and humic-like substances (1.54–1.77 g cm^−3^) from wood burning (Dinar et al. 2006).

#### Incense burning and cooking

Incense burning generated a majority of particles in the accumulation mode with a CMD around 110–150 nm. The total number concentration was up to 2.25 × 10^5^ particle cm^−3^. In the first scan of incense burning, it showed a distinct mode at 30.5 nm, suggesting that some nanoparticles were formed in the first several minutes of burning.

The number size distribution of particles released during incense burning was in agreement with previous studies. Wu et al. (2011) measured particle number size distributions from five types of incense stick and found that four of them had a CMD ranging from 124.1 to 148.9 nm and the other had a CMD of 75.5 nm. A similar study conducted by Ji et al. (2010) reported that the peak number mode of incense smoke was 136 nm which was found to have a larger size distribution in comparison to other combustion processes such as diesel, wood or biomass burning. Unfortunately, the APS sampler had a problem with its inlet during measurement of particle size for incense burning and cooking; hence, this study could not measure the coarse size for incense burning and cooking emissions. In this study, we adopted an effective density of 1.1 and 1.0 g cm^−3^ to convert the aerodynamic to mobility diameter for incense burning and cooking particles respectively based upon Buonanno et al. (2009) and Ji et al. (2010).

Cooking emissions showed a tri-modal distribution with a peak mode at 47.8 nm. After cooking, the peak mode quickly increased to 135.8 nm. The reason is probably related to the ventilation (opened door when people leave kitchen room after cooking activity). During the cooking, a large number of particles were generated with a peak number concentration of 1.57 × 10^6^ particle cm^−3^. In a review of cooking emission, Abdullahi et al. (2013) reported a large fraction of ultrafine particles released during cooking activity with the peak number mode around 20–100 nm.

### Hygroscopic growth factor of particles

#### Hygroscopic growth factor of outdoor and indoor particles when no indoor source was present

**Fig. 3:**
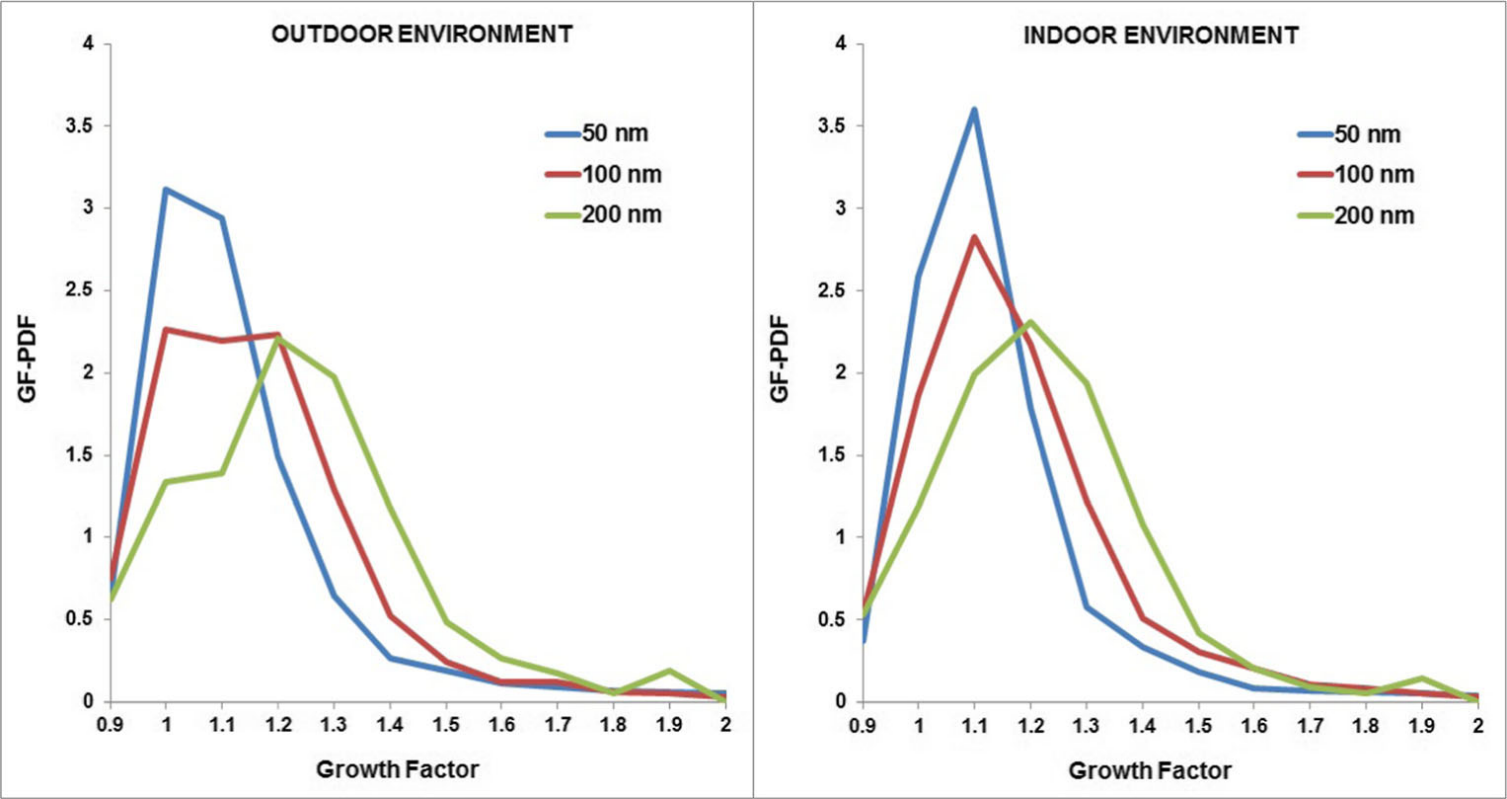
Hygroscopic growth factor probability density function (G_f_-PDF) for outdoor (*left*) and indoor (*right*) particles

The hygroscopic growth factors (G_f_) of outdoor and indoor particles at an initial dry diameter of 50, 100 and 200 nm were measured during periods when no indoor source was present, from 15 to 21 August, 2014. The growth factor probability density function (G_f_-PDF) of each particle size from the outdoor and indoor environments is shown in Fig. 3. For the particle size of 50 and 100 nm, two fractions of nearly hydrophobic (G_f_ ∼1.01–1.11) and less-hygroscopic particles (G_f_ ∼1.11–1.33) were found dominant in both indoor and outdoor environments. For the particle size of 200 nm, the main fractions of particles were less-hygroscopic and more-hygroscopic particles (G_f_ ∼1.11–1.85). Mean growth factors for outdoor particles were 1.15 ± 0.07, 1.17 ± 0.09 and 1.23 ± 0.10 for the particle sizes of 50, 100 and 200 nm, respectively. The mean growth factors for indoor particles were slightly higher than those of outdoor particles with values of 1.16 ± 0.07 and 1.18 ± 0.09 for particles with a diameter of 50 and 100 nm, respectively. The loss of semi-volatile organic constituents due to volatilization or uptake on the indoor wall surface during the penetration from the outdoor to indoor environment could explain the small decrease of the fraction of nearly hydrophobic particles in the indoor environment when no indoor source was present. On the other hand, the mean growth factor for indoor particles with a diameter of 200 nm was 1.22 ± 0.07 which was practically the same as the growth factor for outdoor particles (1.23 ± 0.10).

#### Hygroscopic growth factor of particles generated from indoor sources

**Fig. 4:**
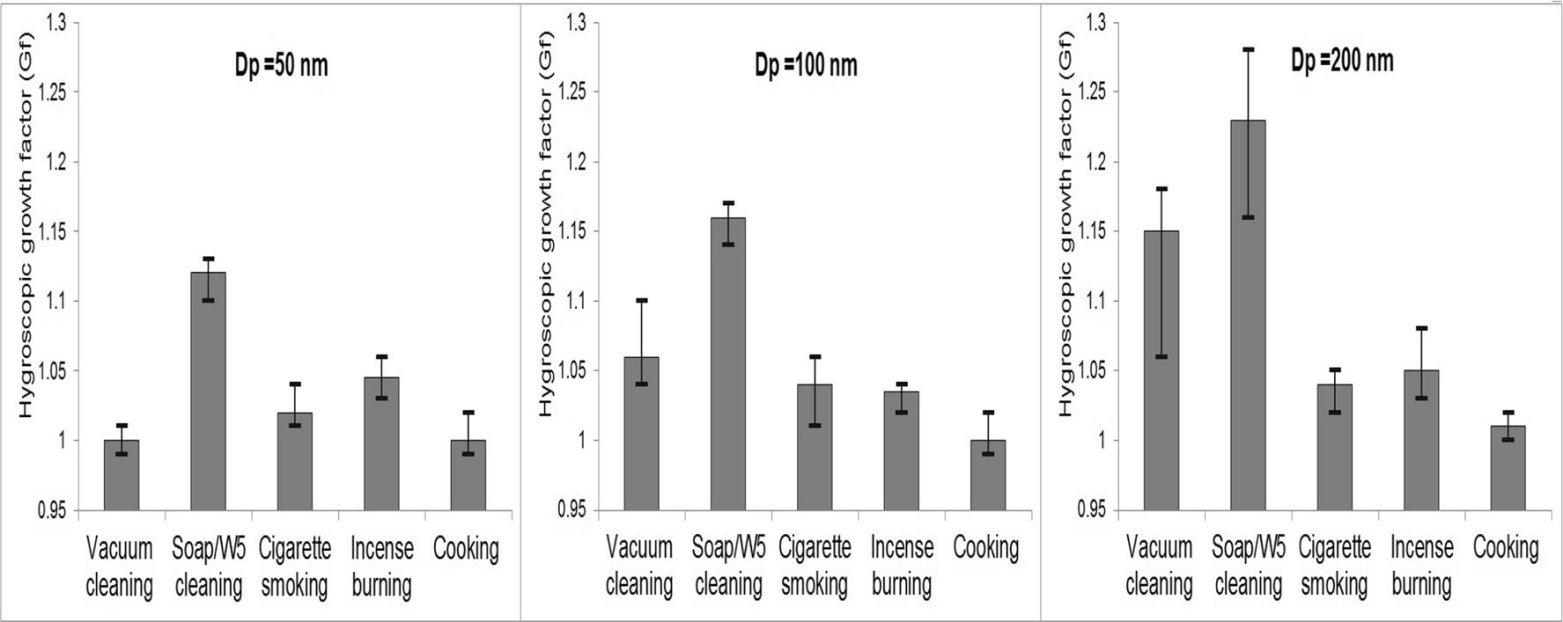
Hygroscopic growth factor of particles generated from different indoor sources

Figure 4 shows the hygroscopic growth factor of particles emitted from five indoor sources at three particle sizes of 50, 100 and 200 nm. Particles generated from vacuum cleaning were found to be “nearly hydrophobic” with an average growth factor (G_f_) around 0.98–1.10 for particle sizes of 50 and 100 nm. This finding is in agreement with the discussed hypothesis that particles emitted from the vacuum cleaner mainly comprise carbon and copper as discussed above. The growth factor of particles of 200 nm was 1.16 which is higher than those for smaller particles due to the mix of particles generated from the vacuum cleaner motor and background.

Particles emitted from Soap/W5 cleaner were found to be “less-hygroscopic” (G_f_ ∼1.12–1.15 for particles at 50 and 100 nm). This finding is consistent with the above suggestion that the ultrafine particles generated by cleaning activities using W5 cleaner predominantly consist of organic compounds such as the products of oxidation of limonene. Virkkula et al. (1999) conducted a measurement of hygroscopic properties of aerosol formed by oxidation of limonene, α-pinene and β-pinene and found that the hygroscopic growth factor was approximately 1.10 at 84 % RH, consistent with our results. The hygroscopic growth factor of particles of 200 nm was 1.22. This high growth factor could also be explained by the mix of particles generated from cleaning activities and background in the large size range, while the majority of particles during this activity were found in the ultrafine size range.

The average hygroscopic growth factors of particles emitted from cigarette smoking were approximately 1.01–1.04 for all sizes of 50, 100 and 200 nm. This hygroscopic growth factor is found slightly higher than diesel combustion (G_f_ ∼1.01), but lower than biomass burning (G_f_ ∼1.04–1.10). This low hygroscopic growth factor is probably due to the chemical properties of particles generated from combustion, which mainly comprise black carbon and organic compounds. Morawska et al. (2005) found a similar count median diameter for both inhaled and exhaled submicron particles, suggesting that the change in particle size after its travel into the lung is not significant. However, the lack of growth of particles cannot be concluded with certainty due to different temperature and humidity regimes between the chamber and the lung. Li and Hopke (1993) found the hygroscopic growth factor of cigarette mainstream and sidestream smoke particles at 200 nm was approximately 1.40–1.42 at RH 99.5 % which was slightly higher than the estimated growth factor value in our study (G_f_ ∼1.3 for Dp = 200 nm at 99.5 % humidity calculated from G_f_ ∼1.04 at 90 % measured by HTDMA).

Similarly, particles emitted from incense burning were found to be nearly hydrophobic with the hygroscopic growth factors ranging from 1.01 to 1.05 for all particle sizes of 50, 100 and 200 nm. A dominance of carbonaceous particles released from incense burning can explain those low hygroscopic growth factors. Wang et al. (2007a) measured the characteristics of air pollutants from incense burning in two temples in Hong Kong and found that the organic and elemental carbon accounted for around 60 % of PM_2.5_ mass, while inorganic ion species only accounted for 12 % of PM_2.5_ mass. Li and Hopke (1993) predicted that the hygroscopic growth factor of particles at 99.5 % RH from the burning of incense was around 1.45 and 1.7 for particle sizes of 100 and 200 nm while the values in our estimation were much lower (G_f_ ∼1.16 and 1.31 for particles of 100 and 200 nm at 99.5 % RH). Li and Hopke (1993) indicated that 8 % errors were found in their estimation of the hygroscopic growth ratio mainly due to the humidity uncertainty in their HTDMA system. Furthermore, variations in particle composition and influences of the local environment may account for differences when measuring the hygroscopic growth factor of combustion aerosols.

Particles generated from cooking activity were found to be nearly hydrophobic (G_f_ ∼1.0–1.02 for all particle sizes of 50, 100 and 200 nm). In our study, we performed the cooking experiments by frying sausages with sunflower oil and toasting bread. This result is consistent with Dua and Hopke (1996), who observed that there was no growth of particles emitted from cooking oils and sweet Italian sausages.

#### Growth factors of particles in the lung

**Fig. 5:**
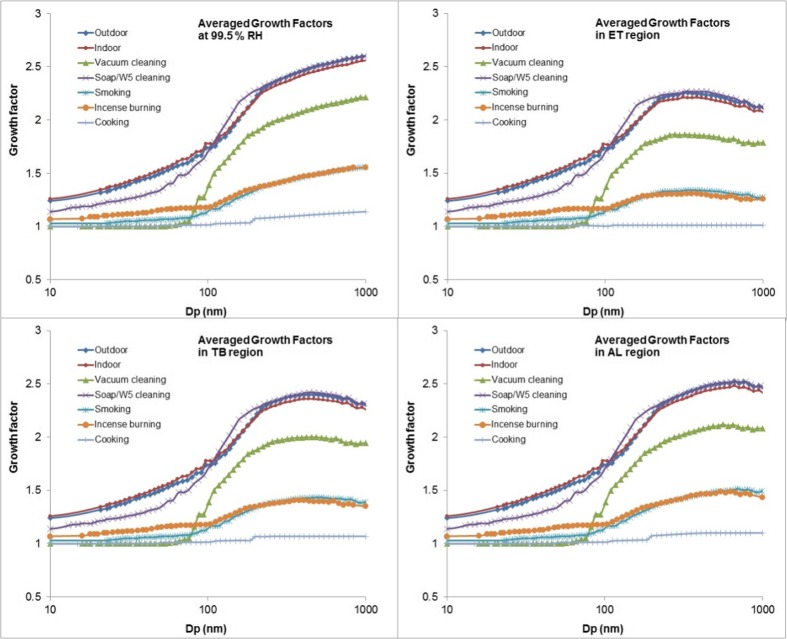
Modelled hygroscopic growth factors at 99.5 % RH and in different regions of the lung

The mean growth factors for particles from outdoors, indoors without indoor sources, and five major indoor sources in their maximum growth at 99.5 % and different regions of the lung are shown in Fig. 5.

As discussed above, the hygroscopic properties of particles arising from different indoor activities show different characteristics. Particles generated from soap/W5 cleaning products show a more hygroscopic tendency than particles from indoor combustion sources. Particles emitted from cooking activities show no significant growth in all sizes. When particles penetrate into the respiratory tract, the particles with diameter below 200 nm can quickly reach their equilibrium size in all lung regions. For particles larger than 400 nm, growth factors in the ET region can be more than 10 % lower than those in the AL region.

### Effects of particles from indoor sources on lung dose of particles

#### MPPD models for different genders and activities

**Fig. 6:**
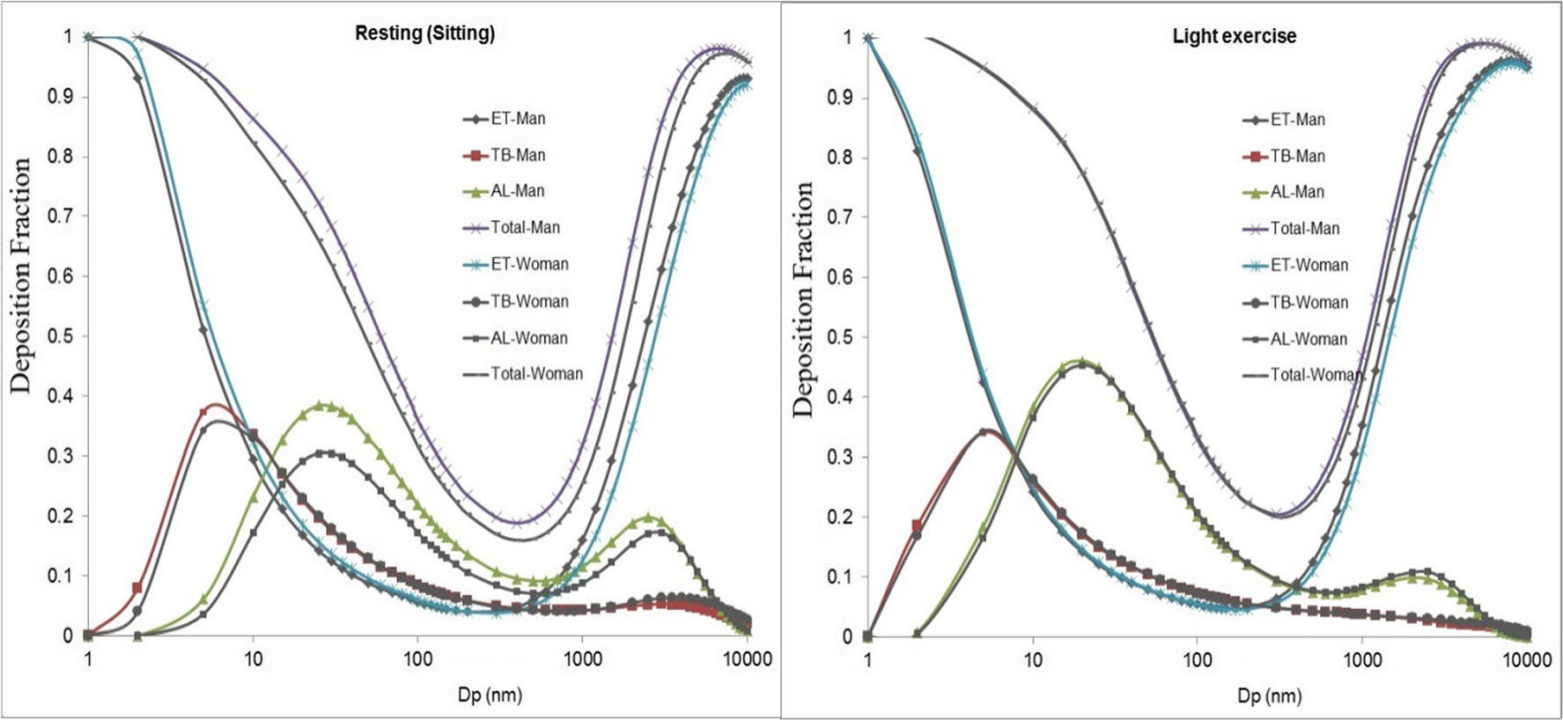
Deposition fraction curve from MPPD model for man and woman in resting and light exercise

Based on the MPPD model, the deposition fraction curves of hydrophobic particles for man and woman in light exercise and resting are shown in Fig. 6. The total deposition fraction was found to be slightly greater for men than women when resting. Specifically, the deposition fraction of ultrafine particles in the AL regions was found greater by up to 1.35 times higher for men compared with women. On the other hand, there was no significant difference between the lung deposition fractions for men and women during light exercise.

#### Regional lung deposition of particles emitted from each source

**Fig. 7:**
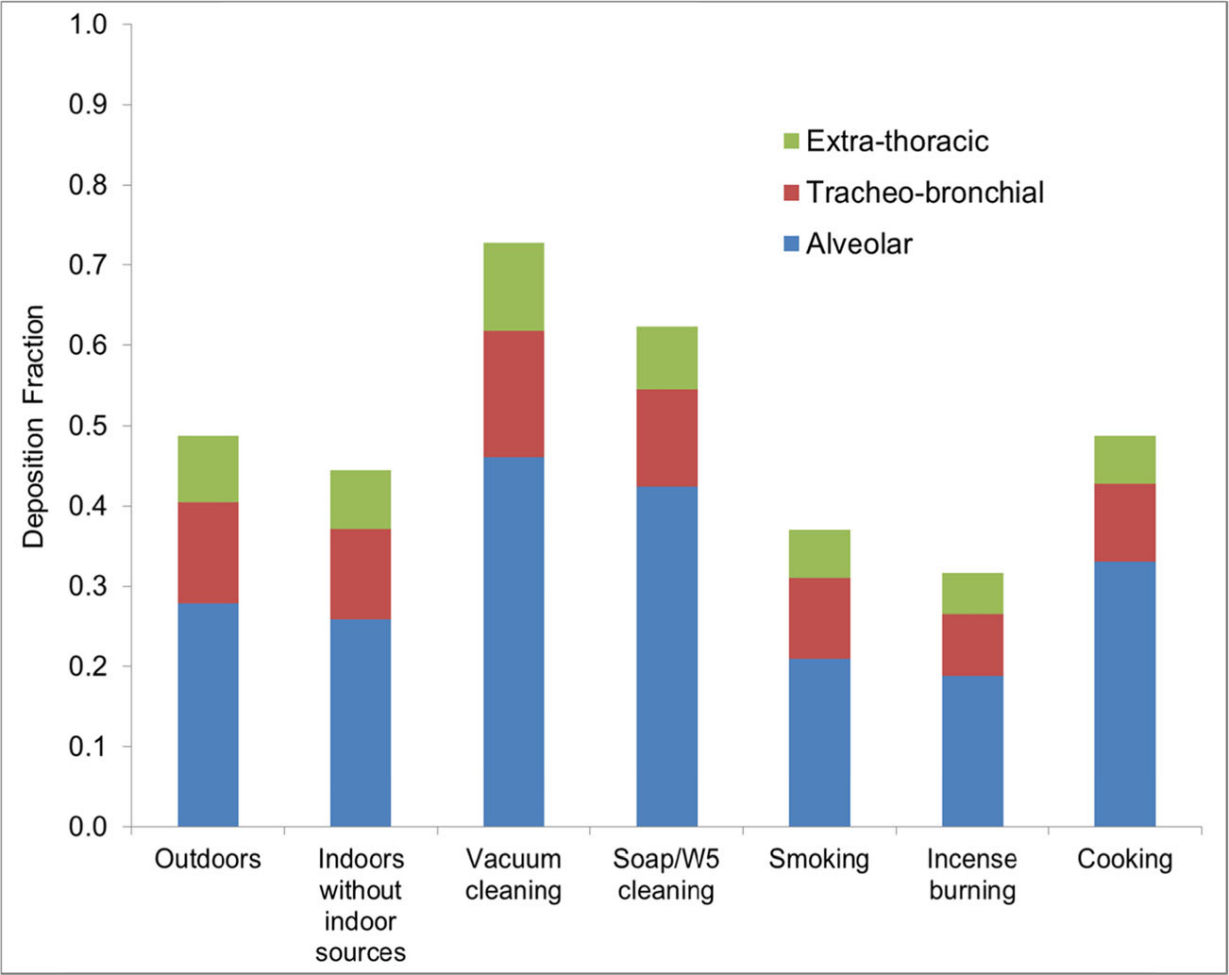
Deposition fraction of particle number in regions of the lung for adults

Total and regional lung deposition fractions of outdoor and indoor particles (with and without indoor source particles) for adults (male and female) by number are shown in Fig. 7. The fractional total lung deposition fraction for outdoor particles and indoor particles (without indoor sources) was 0.49 ± 0.06 and 0.45 ± 0.05. The slightly lower total deposition of particles from the indoor environment compared to the outdoor environment was due to the shift of the outdoor particle number size distribution to a larger size range when they penetrate from outdoor to indoor environments. For both environments, a dominant fraction of particles deposited in the AL region (59.1 %), followed by the TB region (23.9 %). There was only 15.7 % of particle number deposited in the ET region.

For the indoor sources, the lung deposition of particles alters mainly due to the change of size distribution. Up to 66.2 and 72.6 % of total particles deposit in the lung during soap/W5 cleaning and vacuum cleaning. This can be explained by a majority of particles released during cleaning activities being present as nanoparticles which easily penetrate into the deeper region of the lung. In contrast, the lung deposition fraction of particles emitted from indoor combustion sources such as cooking, incense burning or smoking was lower because the main fraction of particle number was found in the larger size range, particularly in the Aitken range for cooking and the accumulation mode for incense burning or cigarette smoke. The total lung deposition fraction was 0.49 ± 0.02, 0.32 ± 0.03 and 0.37 ± 0.03 for indoor particles generated during cooking, incense burning and smoking. For those particles, a predominance of deposited particle number was found in the AL region (56.7–68.1 %), followed by the TB region (19.5–27.0 %). Only 12.2–16.2 % of total deposited particles by number was found in the ET region.

#### Discussion on the contribution of indoor sources to lung dose of aerosols

In order to determine and compare the effects of indoor sources upon human exposure, this study has compared the lung dose rate of particle number in the indoor environment with and without indoor activities. The lung dose of particle number in different regions of the lung (i) within a specific particle size range was calculated based on the following equation (Hussein et al. 2013):

7 $$ Dos{e}_{\mathrm{i}}={V}_E\times D{F}_i\times {C}_N\times \varDelta t $$

where *V*_E_ is the minute ventilation (m^3^ min^−1^); DF_*i*_ is the deposited fraction of particles in the different regions of the human respiratory tract; *C*_*N*_ is the total number concentration (particle cm^−3^); Δ*t* is the exposure time period (minutes). The respiratory tract deposition particle dose rate which is defined as the total particle number deposited in the respiratory system during a specific time period (in this study, Δ*t* was set up to 1 min), can be estimated from:

8 $$ Minute\kern0.5em  dose\kern0.5em  rat{e}_{\mathrm{i}}={\mathrm{V}}_E\times D{F}_{\mathrm{i}}\times {\mathrm{C}}_{\mathrm{N}} $$

*V*_E_ was set at 7.75 × 10^−3^ for sitting and 2.29 × 10^−2^ m^3^ min^−1^ for light exercise calculated by averaging the respective VE values for men and women at two exercise levels (sitting and light exercise) (ICRP 1994).

Table 2 shows the minute dose rate of particles by number for adults in the outdoor and indoor environment (with and without indoor sources). During indoor activities, humans can be exposed to a huge number of particles. For example, a person when cooking could have an exposure of more than 1.76 × 10^10^ particles every minute, which is a thousand times higher than the exposure value for outdoor particles (14.2 × 10^6^ particles). The 1-min dose of cooking particles by number at the light exercise level is equivalent to 18.3 and 6.2 h exposure to outdoor particles at the resting and exercise respiration rate, respectively.

**Table 2:** Lung deposition of particles from outdoor and indoor environments (without and with indoor sources) for adults (averaged deposition fraction for both man and woman)

	Deposition fraction				*V*_E_ (m^3^ min^−1^)	Dose (10^6^ particles min^−1^)			
	AL	TB	ET	Total		AL	TB	TB	Total
Outdoors	0.28	0.13	0.08	0.49	7.75E−03	9.0	4.0	2.6	16.1
Indoors^a^	0.26	0.11	0.07	0.45	7.75E−03	6.6	2.9	1.9	11.4
Vacuum cleaning	0.46	0.16	0.11	0.73	2.29E−02	992.5	338.2	238.0	1568.3
Soap/W5 cleaning	0.42	0.12	0.08	0.62	2.29E−02	1419.3	406.9	258.4	2084.5
Smoking	0.21	0.10	0.06	0.37	7.75E−03	47.1	21.5	13.7	82.9
Incense burning	0.19	0.08	0.05	0.32	7.75E−03	328.7	128.3	86.6	549.5
Cooking	0.33	0.10	0.06	0.49	2.29E−02	11,874.5	3413.3	2159.0	17,630.0

*V*_*E*_ ventilation rate (minute ventilation)

^a^Indoor environment with no indoor sources

Similarly, the total minute lung dose of particles from vacuum cleaning, soap/W5 cleaning, smoking and incense burning could be up to 1.6 × 10^9^, 2.1 × 10^9^, 8.3 × 10^7^, 5.5 × 10^8^ particle min^−1^. Based on Eq. (7), it is clear that the minute lung dose rate of particles strongly depends upon *V*_E_, *DF*_*i*_ and *C*_*N*_. While the total DF_*i*_ for particles from the different indoor sources had a range of 0.32 (for incense burning) to 0.73 (for vacuum cleaning) and the minute ventilation rate for a male adult can range by nearly 6.7 times from 7.5 × 10^−3^ to 5 × 10^−2^ m^3^ min^−1^ (ICRP 1994), the concentration of particles by number was found to show the largest variation from 3.3 × 10^3^ (for indoor environment with no source) to 1.57 × 10^6^ (for cooking emission) particle cm^−3^. This suggests that the concentration level is the main factor controlling the lung dose.

**Fig. 8:**
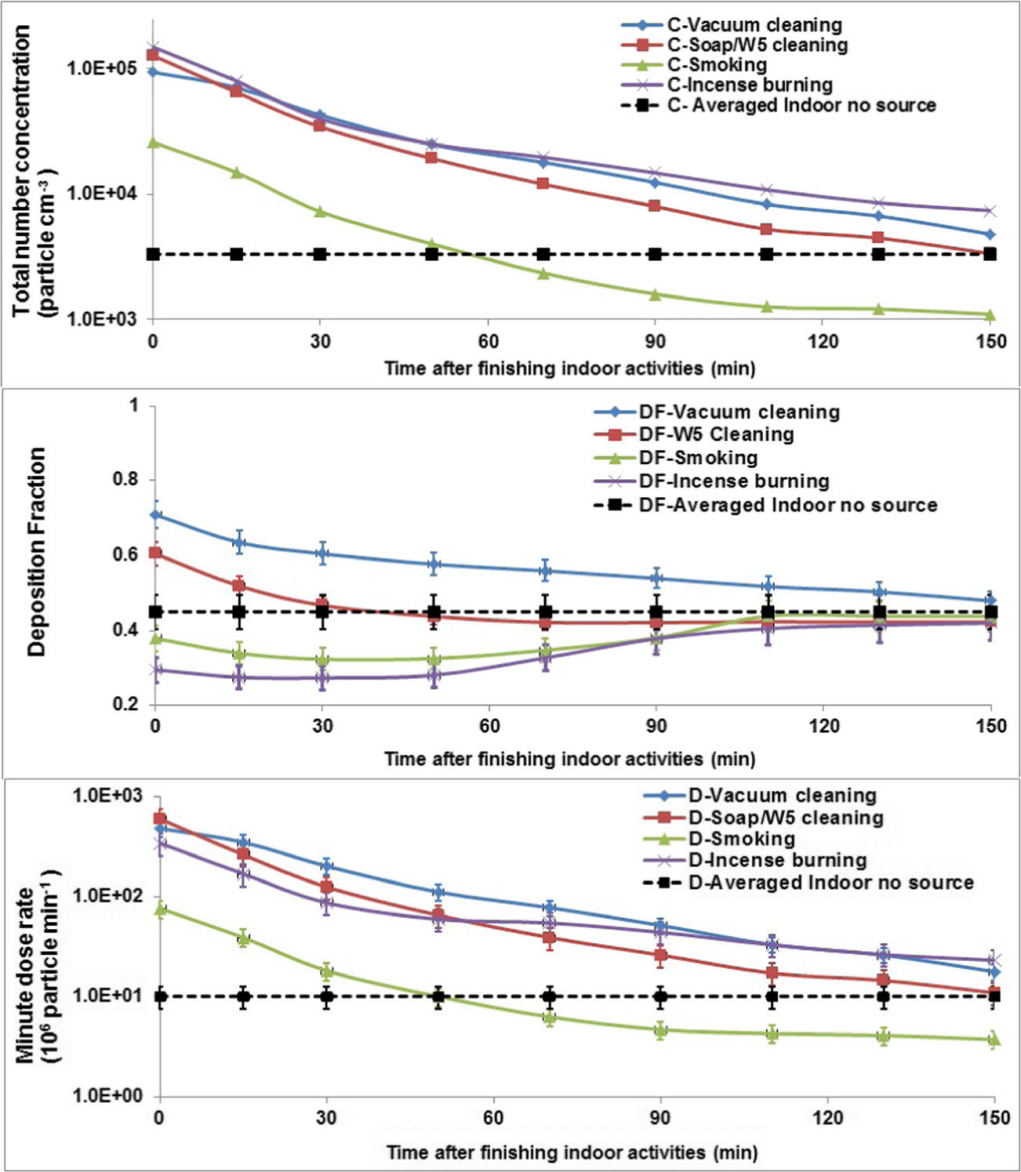
Minute lung dose, concentration and lung deposition fraction of particles after indoor activities

Indoor particles not only contribute a large fraction of human exposure to aerosols during indoor activities, but also after indoor activities. Figure 8 shows the evolution of minute lung dose rate, concentration and lung deposition fraction of indoor particles after finishing indoor activities under low ventilation conditions (closed windows and door after indoor generation). It is found that the total concentration and minute lung dose decrease dramatically after stopping indoor particle generation, but the minute lung dose was still very high within 30 min, especially for cleaning activities. Because of deposition, coagulation and the mixing with outdoor particles due to air exchange, the size was also changed, altering the lung deposition fraction. For cleaning activities, the lung deposition fraction decreased to that of average indoor particles without indoor sources. For incense burning and passive smoking, the lung deposition fraction firstly decreased within around the first 30 min after finishing indoor activities, but then increased to the background level. This can be explained due to the deposition of particles on kitchen surfaces in the first 20–30 min which shifts the particle size distributions to a larger size. Subsequently, the mixing with outdoor particles which have a smaller size than incense burning and smoking aerosols moves the particle number size distribution to the smaller size range, consequently affecting deposition fraction.

#### Uncertainty for minute dose rate calculation

As shown in Eq. (8), the uncertainty for minute dose rate calculation depends upon the variation of minute ventilation (*V*_E_), deposition fraction (DF) and particle number concentration (*C*_*N*_). In this study, the minute ventilation is assumed as a constant value for each activity. Therefore, the uncertainty in the minute dose rate calculation can be estimated from the uncertainties in the lung deposition fraction and particle number concentration. The relative standard error of lung deposition fractions of particles generated from each indoor source mainly depends on the variation of input size distribution and subject difference such as gender and lung structure. Since the MPPD model does not provide estimates of uncertainty, this study could only estimate the errors caused by the variation of particle number size distribution and gender. The estimated relative standard deviations of total lung deposition fraction calculated for vacuum cleaning, soap/W5 cleaning, smoking, incense burning and cooking particles were 2.7, 4.8, 8.1, 9.4 and 4.1 %, respectively.

The uncertainty in particle number concentrations measured for each source depends on the variation of particle number concentrations obtained by each SMPS measurement scan during the period of indoor source generation. The cleaning, smoking, incense burning and cooking experiments were performed during a period of approximately 10, 5, 60 and 20 min, respectively. Each SMPS measurement combined two SMPS scans during 5 min. The relative standard deviation was estimated by dividing the standard deviation by the average number concentration of particles measured during each activity. The estimated relative standard deviation for particle number concentration measurement was 17.4, 9.4, 7.1, 22.3, and 14.4 % for vacuum cleaning, soap/W5 cleaning, smoking, incense burning and cooking measurements. By combination of the relative standard deviation for lung deposition fractions and particle number concentrations, the uncertainty in the minute lung dose rate was estimated as 17.6, 10.6, 10.8, 24.2 and 15.0 %, respectively. Clearly, the uncertainty in minute lung dose rate depends mainly upon the variation of particle number concentration.

## Conclusion

Particles released from indoor activities have many physical properties such as concentration, particle size, particle density and hygroscopicity which are relevant to determining lung deposition. Particles generated from vacuum cleaning and soap/W5 cleaning are mainly distributed in the nano size range while those from incense burning and cigarette smoking were found predominantly in the accumulation mode, and those from cooking activity were found in the Aitken mode. Most of the particles released from indoor sources were nearly hydrophobic, except the particles from soap/W5 cleaning that were less-hygroscopic. Particles from cleaning activities showed very high total lung deposition fraction by number. This was up to 0.73 and 0.66 for vacuum cleaner and soap/W5 cleaning particles, respectively.

Particles are predicted to deposit by number mainly in the AL region, followed by the TB region. This study found that people could be exposed to high aerosol concentrations due to indoor sources. The minute lung dose of particles during indoor source episodes was found to be much greater than the indoor background level without an indoor source. The total minute lung dose rate of particles from vacuum cleaning, soap/W5 cleaning, smoking and incense burning could be up to 1.6 × 10^9^, 2.2 × 10^9^, 7.2 × 10^7^ and 5.4 × 10^8^ particles min^−1^, respectively, while those for average outdoor and indoor background levels were 1.4 × 10^7^ and 1.0 × 10^7^ particles min^−1^. This suggests that indoor sources may make the main contribution to the total lung dose of indoor particles expressed by number.
